# Characteristics and prognostic value of potential dependency genes in clear cell renal cell carcinoma based on a large-scale CRISPR-Cas9 and RNAi screening database DepMap

**DOI:** 10.7150/ijms.51703

**Published:** 2021-03-11

**Authors:** Bowen Shi, Jie Ding, Jun Qi, Zhengqin Gu

**Affiliations:** Department of Urology, School of Medicine, Xinhua Hospital Affiliated to Shanghai Jiao Tong University, Shanghai 200092, P.R. China.

**Keywords:** Clear cell renal cell carcinoma, Depmap database, Dependency, Prognostic value, TCGA.

## Abstract

Background: Large-scale loss-of-function screening database such as Cancer Dependency Map (Depmap) provide abundant resources. Investigation of these potential dependency genes from human cancer cell lines in the real-world patients cohort would evaluate their prognostic value thus facilitate their clinical application and guide drug development.

Methods: A few genes were selected from top clear cell renal cell carcinoma (ccRCC) lineage preferential dependency candidates from Depmap. Their characteristic including expression levels both in normal and tumor tissues and correlations with methylation or copy number, genetic alterations, functional enrichment, immune-associated interactions, prognostic value were evaluated in KIRC cohort from TCGA, GTEx, and multiple other open databases and platforms.

Results: 16 genes were collected from 106 ccRCC preferential candidates and further analyzed including B4GALT4, BCL2L1, CDH2, COPG1, CRB3, FERMT2, GET4, GPX4, HNF1B, ITGAV, MDM2, NFE2L2, PAX8, RUVBL1, TFRC, and TNFSF10. The normalized gene effect scores of these genes varied from different ccRCC cell lines and principal component analysis (PCA) showed their tissue specificity expression profiles. Genetic alteration rates of them were low to moderate (0.7%-13%) in KIRC cohort. CDH2, MDM2, TNFSF10 showed a statistically significant higher level in tumors than normal tissues while PAX8 and FERMT2 were significantly downregulated. Moderate positive or negative correlations were observed in several genes between their expression and relative gene copy number or methylation levels, respectively. Based on the multivariable COX regression model adjusted by critical clinical variables revealed the expression of GET4 (p=0.002, HR=1.023 95%CI 1.009-1.038) and CRB3 (p<0.001, HR=0.969 95%CI 0.960-0.980) were independent predictive factors for overall survival in KIRC cohort.

Conclusions: A dependency gene validated in cell lines didn't directly represent its role in corresponding patients with same histological type and their prognostic value might be determined by multiple factors including dependency driven types, genetic alteration rates and expression levels. GET4 and CRB3 were the independent prognostic factors for ccRCC patients. CRB3 seemed like a potential broad tumor suppressor gene while GET4 might be a ccRCC preferential dependency gene with a ligandable structure.

## Introduction

Clear cell renal cell carcinoma is the most common form of kidney cancer, and its 5-year survival rate drops to less than 10% for patients with metastatic forms of the disease in the United States 1. The tumor suppressor gene VHL could be inactivated by somatic mutation, deletion or hypermethylation in most ccRCC patients is and associated with tumorigenesis at the earliest stage of the disease 2. Loss and impairment of VHL lead to the stabilization of hypoxia-inducible factor proteins that upregulate the expression of cellular proliferation and angiogenesis genes, such as vascular endothelial growth factor, and elevate the corresponding protein levels, and these findings directly prompt the application of tyrosine kinase inhibitors (TKIs), a revolutionary treatment for ccRCC patients 2, 3. However, although driven mutations influence the clinical progression and outcomes of patients, other essential genes inside tumors could play a critical role and orchestrate the invasion, proliferation, and metastasis of cancer. Recently, large-scale screening projects such as DepMap based on RNA interference silencing and CRISPR-Cas9 knockout techniques have provided an unprecedented comprehensive picture of potential dependency genes whose expression or existence is critical for the survival or proliferation of a hundred cancer cell lines, and these identified tumor vulnerabilities could facilitate the development of potential therapeutic targets 4, 5. A previous study showed that Werner syndrome ATP-dependent helicase (WRN) is a candidate screened from dependency projects and is selectively essential in microsatellite instability (MSI) models both *in vitro* and *in vivo* 6. However, MSI is rare in some tumor types, such as prostate, melanoma and kidney cancer, and even cell lines with MSI from these tissue sites are also not dependent on WRN 7. Furthermore, investigation and validation of these potential dependency genes in patients for their prognostic relevance would advance their translational value and promote their potential clinical application. Assessment of context-specific fitness genes that may have low potential toxicity compared to pancancer core fitness genes and their tractability revealed hundreds of potential targets, but their applications need further study 7. Therefore, subtype-preferentially essential genes from ccRCC cell lines were collected from the DepMap website, and their characteristics and prognostic value were evaluated through multiple aspects using several online analysis platforms. Normal tissue expression profiles in the GTEx database and kidney renal clear cell carcinoma (KIRC) cohort (n=537) in TCGA database were analyzed.

## Materials and Methods

### DepMap

The Cancer Dependency Map (https://depmap.org/portal/) is an accessible website 4 based on large-scale multiomics screening projects, including Cancer Cell Line Encyclopedia (CCLE) 8, a computational algorithm model analyzing the results of three massive RNAi screening datasets called DEMETER2 9; the PRISM Repurposing dataset using pooled-cell line chemical-perturbation viability to screen small molecules 10; and the Achilles Project based on genome-scale CRISPR-Cas9 knockout screens 5. The top 10 preferentially essential genes from ccRCC cell lines identified by two projects on the DepMap database were collected. A gene that could be found in both screening method datasets across ccRCC cell lines or more than one ccRCC cell line was selected to form a signature gene set with potential prognostic value for further analysis. The gene effect scores of each gene in this set, DEMETER2 (RNAi) and CERES (CRISPR), which were derived from screening experiments, were also collected from DepMap. Simply, these two scores evaluated the effect size of knocking out or knocking down a gene while normalizing expression against the distribution of pan-essential and nonessential genes 4, 5. Negative scores represent that the cell line would grow slower, while positive scores show that the cell line would grow faster after experimental manipulation. Normally, the cutoff would be set as -0.5 for a score that represents obvious depletion in a cell line, and positive scores should be cautiously interpreted since a fitness advantage could be caused by random events 5.

### GEPIA2

Gene expression profiling interactive analysis 2 (GEPIA2, http://gepia2.cancer-pku.cn/) is a web server for expression profiling and user-friendly interactive analysis based on the GTEx and TCGA databases (provisional) 11. In the present study, principal component analysis (PCA), gene expression profiles, and comparisons between tumor and normal tissues containing these genes were performed on GEPIA2. In addition, the pancancer prognostic value and its specific correlation within KIRC were measured by GEPIA2. The maps of overall survival (OS) and disease-free survival (DFS) were measured using the Mantel-Cox test across cancer types with a significant false discovery rate (FDR) set at FDR<0.1. Additionally, Kaplan-Meier (KM) curves with log-rank tests were generated. One-way ANOVA was used to explore the correlation between pathological stage and gene expression. A p-value < 0.01 and log2-fold change > 1 were considered significant for comparisons between tumor and normal tissues using t-tests, and gene expression was usually log2(TPM + 1) transformed.

### cBioPortal

The cBioPortal for Cancer Genomics (http://cbioportal.org) was specifically designed to provide a web resource for exploring, analyzing, and visualizing cancer data without barriers 12. Portals are a tool to analyze multidimensional molecular profiling, including genetic, epigenetic, and proteomic features, and thereby accelerate medical translational research 12. Genetic alteration of potential dependency genes and frequently altered genes in the KIRC dataset (TCGA, provisional) was obtained from cBioPortal. Significant expression events were defined as a z score threshold of ±2.0 for mRNA expression (RNA-Seq V2 RSEM) and protein expression (RPPA). The correlation between methylation and the expression level of potential dependency genes was also collected from this website with both Spearman and Pearson variables.

### GSCALite

Gene Set Cancer Analysis (GSCALite, http://bioinfo.life.hust.edu.cn/web/GSCALite/) is an interactive web server, especially for gene sets analyzed based on TCGA and GTEx datasets, and it provides a flexible manner for research to analyze the complex correlation between a gene set and single or multiple cancer types 13. Normal tissue expression, comparison between normal and paired tumor tissues, pathway activity, and miRNA regulation of the potential dependency gene set was obtained from GSCALite using the analysis modules of TCGA and GTEx. The P-value of the comparison was analyzed using a t-test and adjusted by FDR. The genes with fold change (FC > 2, tumor vs normal) and significance FDR (< 0.05) are shown on plots for 5 cancer types in TCGA, including KIRC, kidney renal papillary cell carcinoma (KIRP), kidney chromophobe (KIRC), bladder urothelial carcinoma (BLCA), and prostate adenocarcinoma (PRAD). Cancer-related pathway activity was obtained from GSCALite according to the default variables based on the reversed-phase protein array of TCGA. Analysis of the miRNA regulation network on GSCALite was based on multiple open databases, and the association between paired miRNA and mRNA expression was performed using Pearson's correlation method with a t-distribution test adjusted by FDR and genes with FDR≤0.05. R<0 will be shown on plots 13.

### Functional enrichment databases

GeneMANIA (http://www.genemania.org) is a flexible user-friendly website designed to analyze functions for a gene or gene list based on numerous datasets and interactions from multiple sources, and it can visualize the relationship of proteomic and genetic interactions, coexpression, and related pathways for submitted genes 14. GeneMANIA could also find functionally similar genes using weighted predictive values 14. In the present study, potential dependency genes derived from DepMap were analyzed using the default settings. Metascape (http://metascape.org) provides a biologist-oriented resource to interpret critical components of pathways and protein complexes associated with queried genes 15. In our study, the default analysis module was utilized with a p-value cutoff of 0.01 based on the Gene Ontology (GO) and Kyoto Encyclopedia of Genes and Genomes (KEGG) pathways to further verify the enrichment of 16 selected dependency genes. The website of g:Profiler (https://biit.cs.ut.ee/gprofiler) is another freely available tool to provide reliable service on the basis of simultaneously updating high-quality data from multiple sources 16. All 106 dependency-associated candidate genes were submitted, and functional enrichment of these genes was evaluated using the cumulative hypergeometric test with the default settings and highlighted for the results of interest.

### Immune-associated databases

Tumor Immune Estimation Resource (TIMER, https://cistrome.shinyapps.io/timer/) is a useful tool for the investigation of the molecular characteristics between tumor-infiltrating cells and the host immune system 17. It provides a user-friendly web server for integrated analysis of a wide spectrum of factors associated with the infiltration of immune cells, and the "Gene" analysis tub was used to explore the correlation between 16 genes and different tumor-infiltrating immune cells. TISIDB (http://cis.hku.hk/TISIDB/index.php) is an integrated tumor-immune interaction repository for users to explore the correlation between submitted genes and immune features through literature mining and genomic data analysis 18. Two datasets related to urological cancer 19, 20 on this web portal were selected to evaluate the correlation between immunotherapy responses and the expression of 16 potential dependency genes in our study.

### Quantitative real-time polymerase chain reaction analysis (qPCR)

Total RNA was extracted from RCC cell lines (786-O, SW839, A498) and human embryonic kidney 293T (HEK 293T) using RNAeasy™ Animal Long RNA Isolation Kit with Spin Column (Beyotime Biotechnology Co., Ltd., Shanghai, China). PrimeScript™ RT Master Mix (Takara Biotechnology Co., Ltd., Shiga, Japan) was used to perform reverse transcription. Hieff® qPCR SYBR® Green Master Mix (Yeasen Biotechnology Co., Ltd., Shanghai, China) was used to perform qPCR followed by manufacturer's instructions. The following primers synthesized by Sangon Biotech Co., Ltd. (Shanghai, China) were used: GAPDH forward, 5'-AAG GTG AAG GTC GGA GTC AAC-3' and reverse, 5'-GGG GTC ATT GAT GGC AAC AATA-3'; β-actin forward, 5'-CCT CTC CCA AGT CCA CAC AG-3' and reverse, 5'-GGG CAC GAA GGC TCA TCA TT-3'; CRB3 forward, 5'-ATG AGA ATA GCA CTG TTT TGC C-3' and reverse, 5'-GAT GAT AGC AGT GAT GGC TTC T-3'; GET4 forward, 5'-CAC CAG ATG TAC CGG ACC CT-3' and reverse, 5'-GGC TGA ACA CTT TAG CCA GAT T-3'. Raw data were analyzed using the comparative Ct method and GAPDH and β-actin were used as internal controls.

## Results

### Selection of potential dependency genes and their characteristics

**Figure 1:**
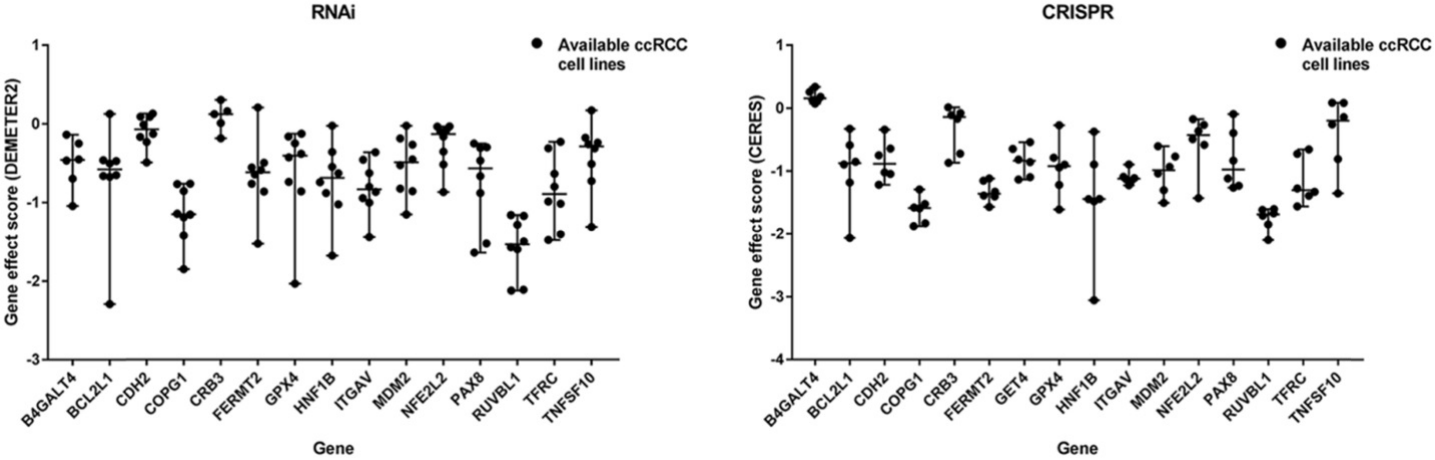
Gene effect scores of genes from RNAi and CRISPR/Cas9 screens.

**Figure 2:**
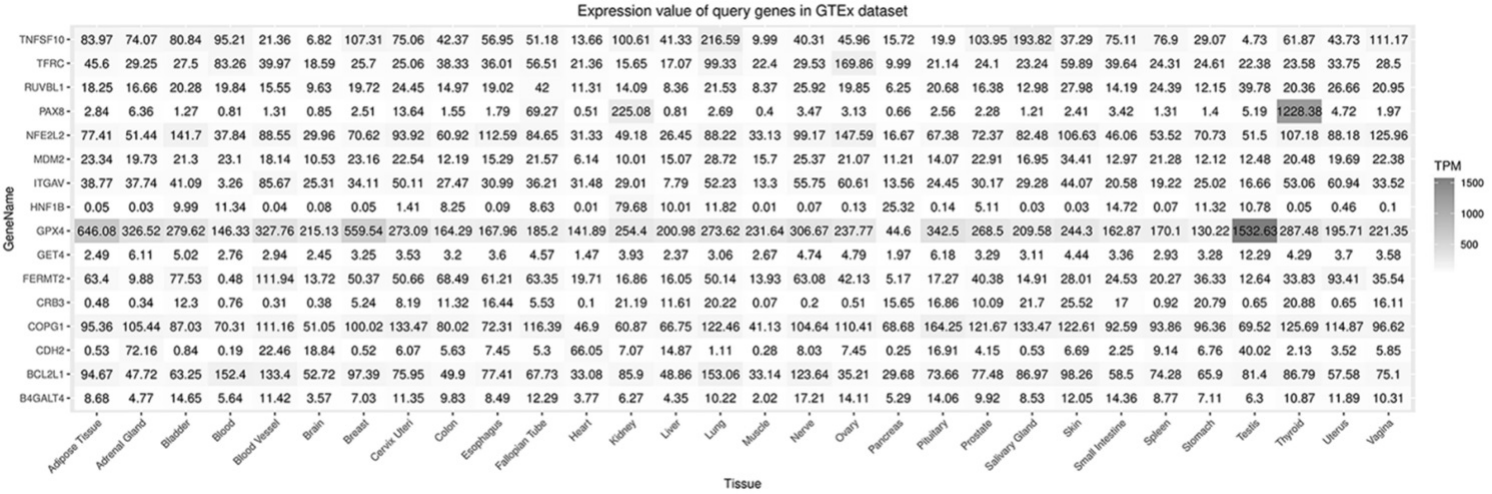
Expression of selected genes in normal tissues.

A flow diagram of data acquisition and analysis is detailed in Supplementary Figure 1. In total, 8 cell lines with available gene effect scores from at least one screening method from all 16 ccRCC cell lines with confirmed disease and lineage subtypes were selected. Overall, 16 genes were chosen from 106 candidate genes according to whether they exist in more than one ccRCC cell line or both screening datasets across cell lines. The gene effect scores of these genes are illustrated in Fig. 1, and one of the GET4 genes had only an RNAi score. The distribution of gene effect scores between genes was different. For example, HNF1B, BCL2L1, TNFSF10, and PAX8 showed a wide range of scores across cell lines, while B4GALT4, CDH2, CRB3, and RUVBL1 represented an obviously narrow range. On the other hand, each gene exhibited different tendencies as potential tumor suppressor genes or oncogenes according to the definition of the gene effect scores. Theoretically, RUVBL1 and COPG1 showed a strong tendency towards oncogenes, while CRB3 remained a nonessential or tumor suppressor gene. The inconsistency of scores among different lineage cell lines within the same ccRCC tissue site and subtype indicates potentially different underlying mechanisms and features of cell lines. The expression profiles of these 16 genes in normal and tumor tissues of different organ systems are illustrated in Fig. 2. In general, these genes are broadly expressed across organ sites in both normal and tumor tissues, but their expression patterns and levels are quite different. GPX4 had the highest baseline expression level among these genes in normal tissues and was extremely highly expressed in the testis. HNF1B had the lowest baseline expression level but was significantly highly expressed in the kidney. Most of these genes have a relatively high or moderate expression level in the kidney compared to other organs. Similar expression patterns could be found in tumor tissues across TCGA datasets (data not shown).

**Figure 3:**
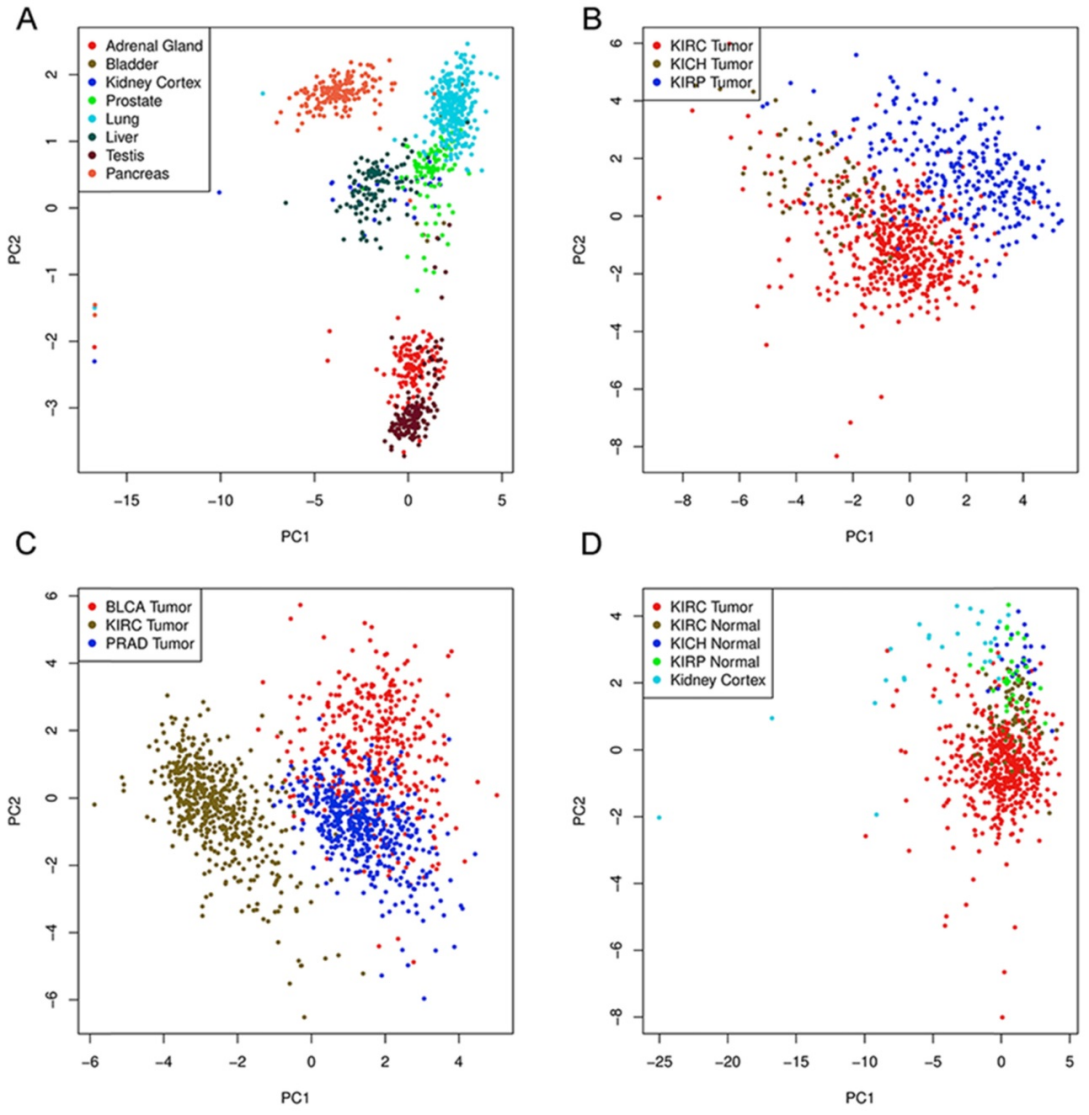
Principal component analysis based on expression of genes.

**Figure 4:**
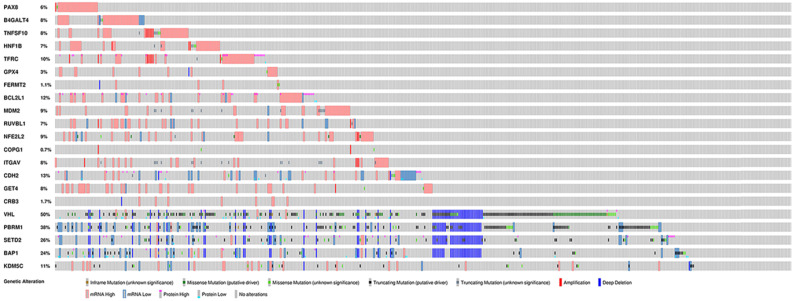
Genetic alterations of selected genes in KIRC cohort.

To further explore the potential tissue or tumor specificity, these 16 genes were considered a signature gene set, and PCA was performed among multiple comparison groups (Fig. 3A-D). First, the results based on several normal tissues show that this set of genes could distinguish samples from different sites. PCA also revealed that this signature set could mainly discriminate ccRCC from other renal cell subtypes or other urological cancers. Finally, PCA of ccRCC, tumor-adjacent normal tissue of three renal carcinomas and normal kidney cortex tissues showed the discriminative ability of the gene set between tumor and normal tissues. Next, the genetic alteration of these genes in the KIRC cohort was evaluated, and the most frequently altered genes, such as VHL and PBRM1, were used as references (Fig. 4). Briefly, more than half of the genes had a moderate alteration rate (≥ 8%), and others showed a low rate of genomic change (0.7%-3%). Among them, BCL2L1 had the highest rate, while COPG1 had the lowest rate, and a high mRNA expression level beyond the z-score thresholds was the predominant event. Notably, genetic alterations of VHL and PBRM1 were common within ccRCC patients, as expected. Additionally, the expression profiles of most genes were weakly (Spearman coefficient <0.4) positively correlated with their relative gene copy number, while B4GALT4, COPG1, and FERMT2 had moderate correlations with Spearman coefficients greater than 0.4 (Supplementary Table 1).

**Figure 5:**
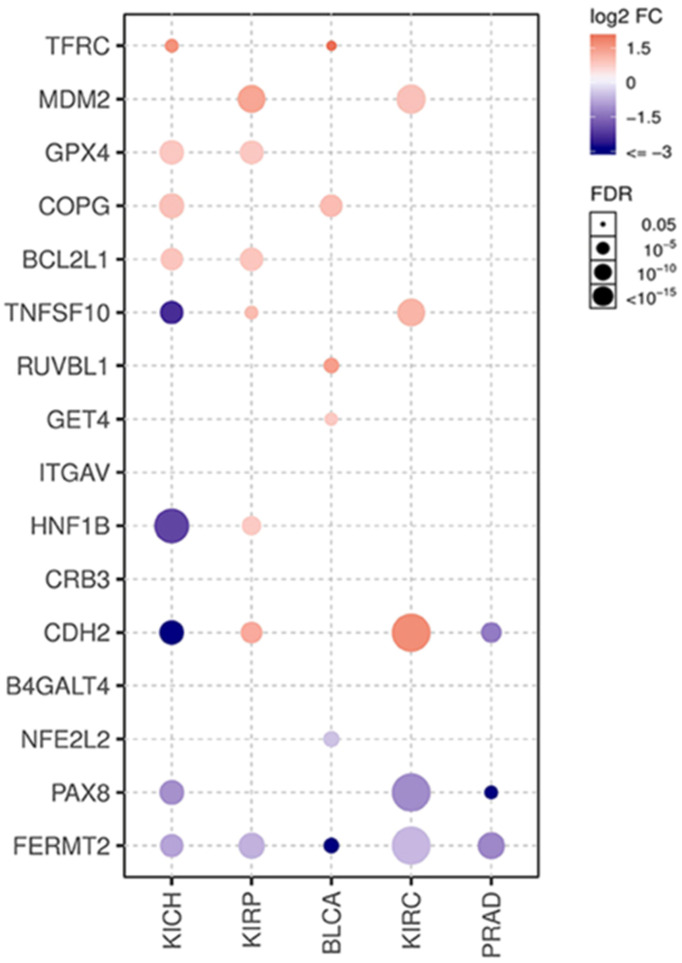
Different expression in paired tumor and normal tissues.

In addition, the expression levels of each gene were compared between tumor and unpaired adjacent normal tissue in the KIRC cohort (Supplementary Figure 2). Interestingly, only CDH2 and MDM2 showed significantly higher levels in tumors than in normal tissues. B4GALT4, BCL2L1, COPG1, CRB3, GET4, and TFRC showed almost the same expression levels between tumor and normal tissues. TNFSF10 and GPX4 showed higher expression levels in tumor tissue, while RUVBL1, PAX8, NFE2L2, HNF1B and FERMT2 showed lower levels, but these differences did not reach statistical significance. Then, 72 pairs of patient-matched tumor and normal tissue samples in the KIRC dataset were used to validate the dataset, and comparisons between paired samples from other urological cancers were also illustrated (Fig. 5). The expression of CDH2, MDM2 and TNFSF10 was significantly upregulated in tumor tissue, while expression of PAX8 and FERMT2 was significantly downregulated. These findings were largely consistent with previous comparison results of the unpaired KIRC samples. Finally, we conducted a correlation analysis between the gene expression and methylation levels (beta-value). Among genes with available methylation data, CRB3 and TNFSF10 showed moderately negative correlations, and the other 12 showed weakly negative correlations (Supplementary Table 1).

### Functional analysis

**Figure 6:**
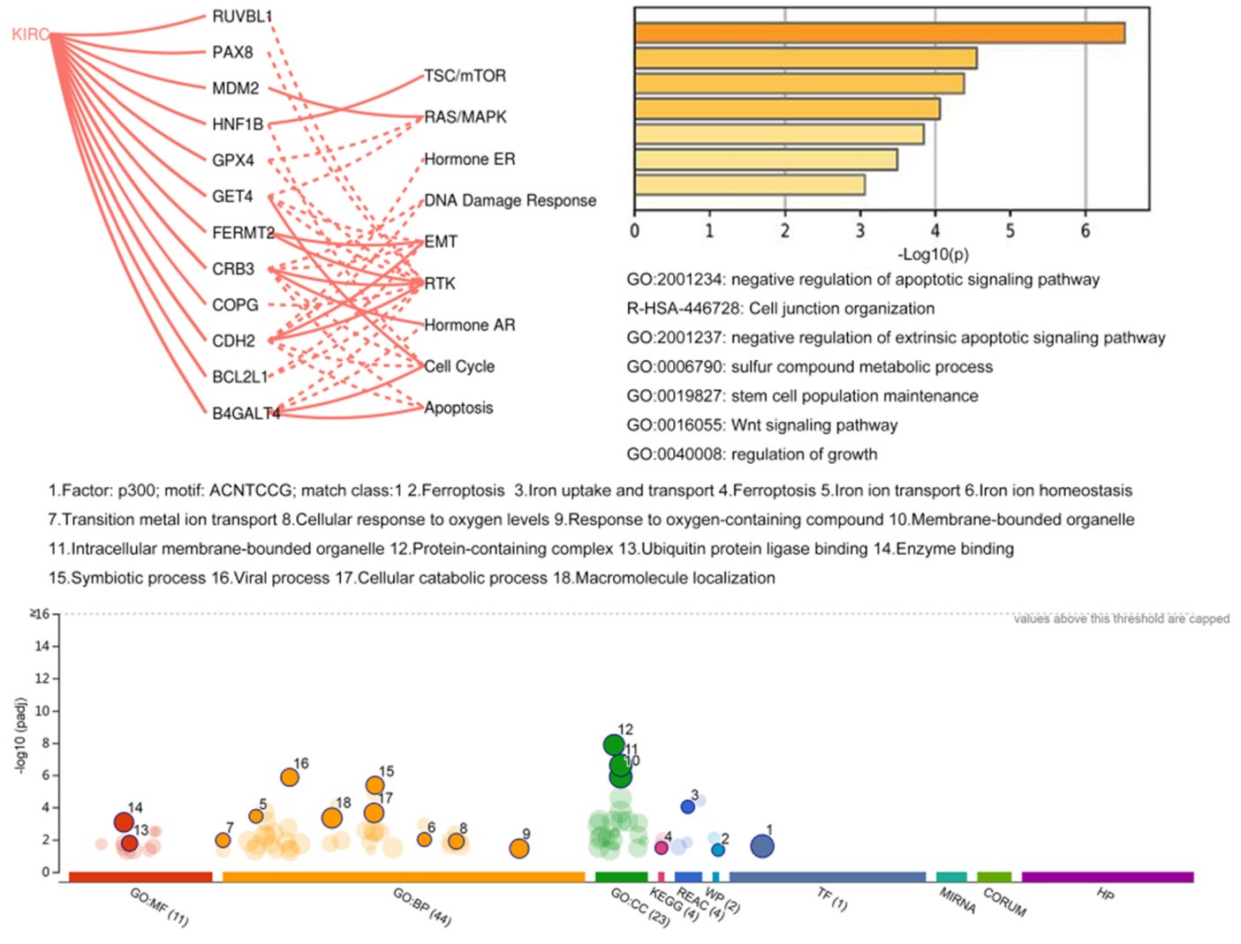
Functional analysis of potential dependency genes.

The network of coexpression and physical and genetic interactions among 16 genes was constructed on the GeneMANIA website with their correlated genes (Supplementary Figure 3). Then, Metascape was utilized to perform GO and KEGG enrichment analyses (Fig. 6). The results showed that these genes were mostly correlated with the apoptotic signaling pathway. In addition, genes with available protein level data in TCGA project were analyzed using the GSCALite platform. The regulatory correlations among 12 genes and several critical pathways derived from the TCGA context are illustrated in Fig. 6. For example, the most affected RTK pathway inside this map could be activated by FERMT2, CRB3, and CDH2, and it could also be inhibited by RUVBL1, PAX8, GPX4, COPG, and B4GALT4. The pathway associated with the cell cycle could be activated by GET4 and B4GALT4 and inhibited by HNF1B, CRB3, and CDH2. Then, the regulatory network of miRNAs (data not shown) based on the GSCALite platform revealed that a few miRNAs could affect multiple objects among these signature genes, such as target genes of hsa-miR-548x-3p, including BCL2L1, ITGAV, FERMT2, NFE2L2, and TNFSF10. Hsa-miR-340-5p also has multiple targets among these genes, including ITGAV, NFE2L2, TNFSF10, and B4GALT4. Moreover, we performed functional enrichment analysis of all 106 candidate genes in the gProfiler database (Fig. 6). Of note, p300 seems to be a key transcription factor among these genes. Additionally, ferroptosis and metabolic regulation of iron ions were widely associated with these potential dependency genes.

### Immune-associated analysis

**Figure 7:**
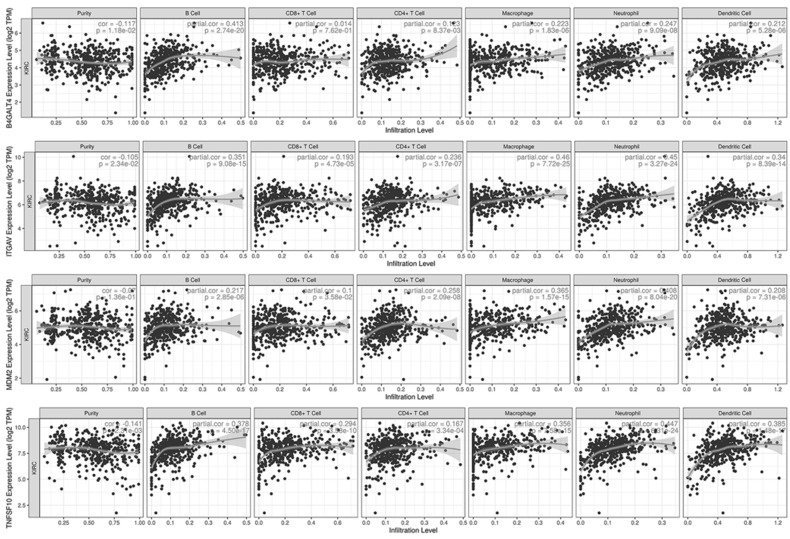
Immune-associated analysis.

Sixteen selected genes were submitted to the TIMER database to explore their correlations with tumor purity and 6 tumor-infiltrating immune cells. A gene with at least one moderate correlation (Cor≥0.4 and p-value<0.001) with any immune cell was considered to have a potential influence on tumor-immune interactions (Fig. 7). According to the analysis results, B4GALT4 was positively correlated with B cells (Cor=0.413), while ITGAV was positively correlated with macrophages (Cor=0.460) and neutrophils (Cor=0.450); in addition, B4GALT4 also had a weak correlation with B cells (Cor=0.351) and dendritic cells (Cor=0.340). MDM2 was positively correlated with neutrophils (Cor=0.408) and macrophages (Cor=0.365), as was TNFSF10 (neutrophils (Cor=0.447) and macrophages (Cor=0.356)). In addition, these signature genes were submitted to the TISIDB web portal to evaluate correlations between their expression level and immunotherapy responses in clear cell renal cell carcinoma and urothelial carcinoma cohorts (Supplementary Figure 4). In the small-size ccRCC cohort, only CRB3 showed a potential difference between responders (n=4) and nonresponders (n=8), but the difference did not reach statistical significance. In another large urothelial carcinoma cohort, several genes showed significant differences between responders (n=68) and nonresponders (n=230). COPG1, RUVBL1, and TFRC were significantly highly expressed in responsive patients, while the expression levels of GPX4, ITGAV, NFE2L2, and PAX8 were significantly lower in responders than in nonresponders.

### Prognostic value of the selected genes

**Figure 8:**
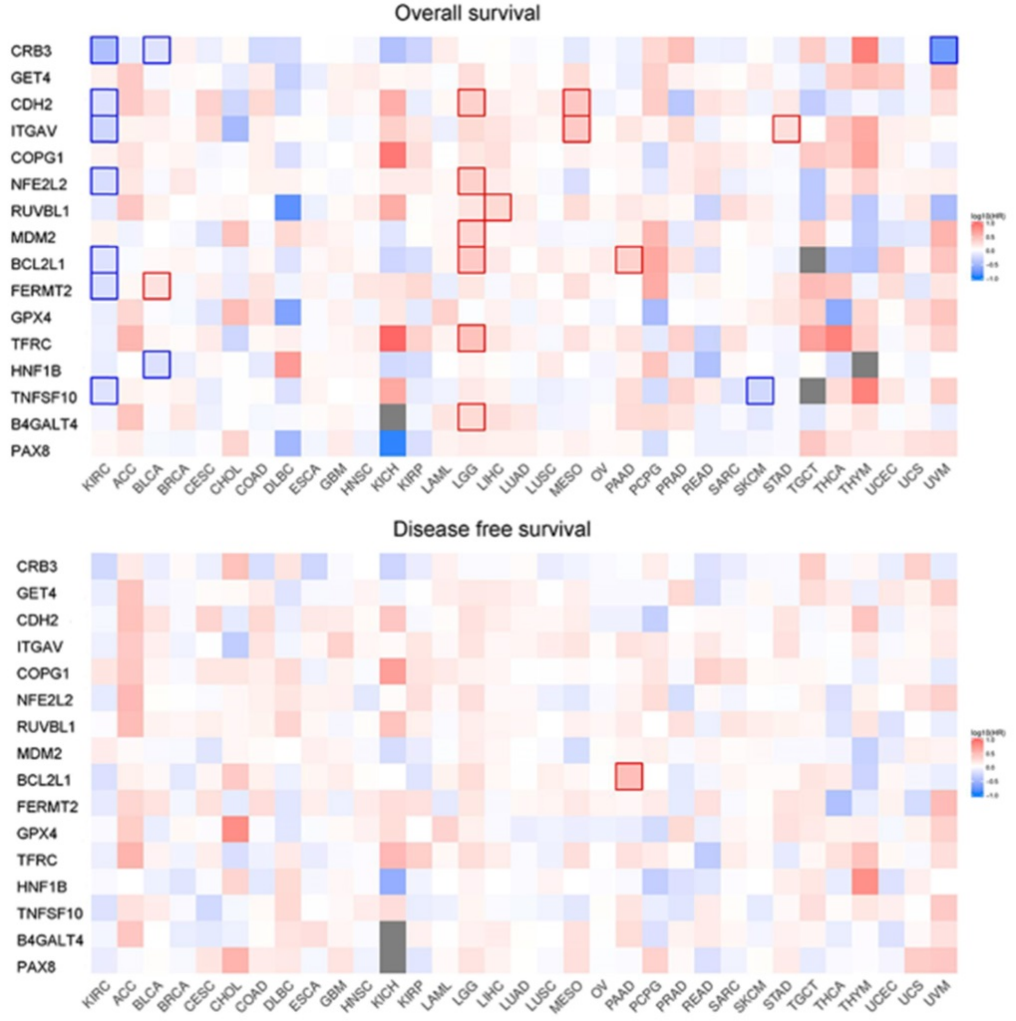
Survival map of OS and DFS.

**Figure 9:**
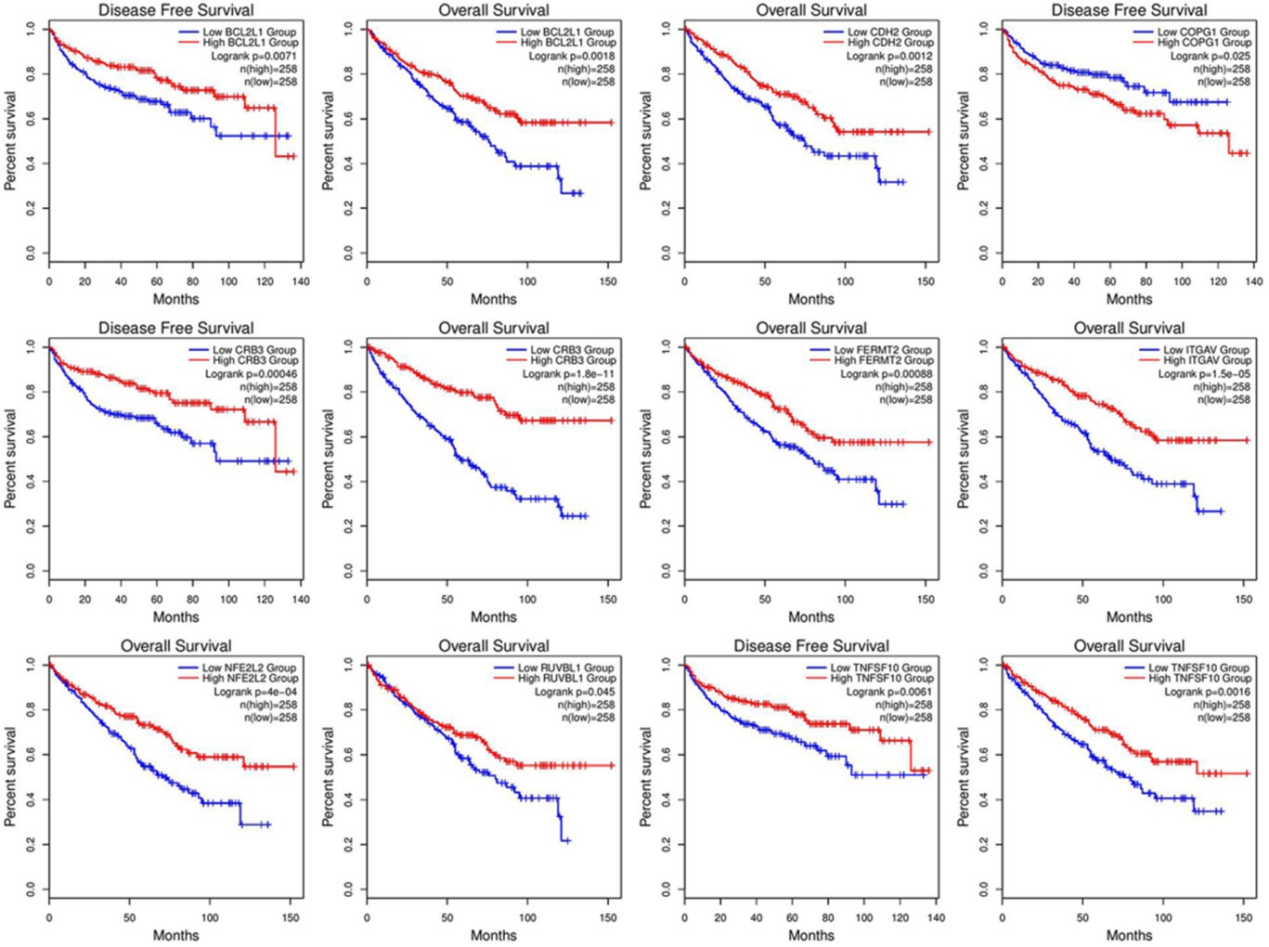
KM curve with log-rank test.

Correlation analysis of overall survival and disease-free survival with these genes across 33 TCGA datasets was performed on GEPIA2 with FDR<0.01 as the significance level (Fig. 8). Low expression levels of CRB3, CDH2, ITGAV, NFE2L2, BCL2L1, FERMT2, and TNFSF10 showed a tendency to be risk factors for decreased OS. After p-value adjustment (FDR), none of the gene expression levels were significantly correlated with DFS in the KIRC cohort (Fig. 8). It is worthwhile that these signature genes have almost completely opposite prognostic value in several tumors, such as low-grade glioma, adrenocortical carcinoma or liver hepatocellular carcinoma. Then, KM curves of OS or DFS among signature genes reaching the significance level are illustrated in Fig. 9. Highly expressed BCL2L1, CRB3, and TNFSF10 in ccRCC patients showed a favorable prognosis in both OS and DFS. High expression levels of CDH2, FERMET2, ITGAV, NFE2L2, and RUVBL1 would be better prognostic predictors for OS. The low expression level of COPG1 would be a prognostic factor with better clinical outcomes (DFS). Furthermore, univariable and multivariable Cox proportional hazard models of overall survival were conducted to identify independent prognostic factors with the adjustment of clinical stages, sex and age in the KIRC cohort (Table 1). In total, 526 ccRCC patients in the KIRC cohort with available complete clinical information, expression levels of 16 genes, and survival data were analyzed. In the univariable Cox analysis, clinical tumor stage, age, and the expression levels of NFE2L2, ITGAV, GET4, FERMT2, CRB3, CDH2, and BCL2L1 were prognostic factors for OS. In the multivariable analysis, tumor stage (stage III vs stage I, HR=2.057, 95% CI 1.355-3.124), especially metastatic stage (stage IV vs stage I, HR=5.518, 95% CI 3.707-8.214), was still a strong prognostic predictor, as expected, and age (p<0.001, HR=1.032, 95% CI 1.017-1.047), expression of GET4 (p=0.002, HR=1.023, 95% CI 1.009-1.038) and CRB3 (p<0.001, HR=0.969, 95% CI 0.960-0.980) were also independent predictive factors. A predictive model of overall survival based on multiple factors including tumor stage, age, and the CRB3 and GET4 expression levels was constructed, and the corresponding ROC curves with time-dependent area under the curve (AUC) values are illustrated in Supplementary Figure 5. Uno's C-index (calculated by the R package "survC1"), which is similar to the AUC value, was 0.68 (1-year), 0.62 (2-year), and 0.59 (3-year).

In addition, we explored the roles of CRB3 and GET4 in pancancer screening on the "Data explorer" module of the DepMap database, including RCC and non-RCC, MSI and MSS, and primary and metastatic tumor cell lines (Supplementary Figure 6). Compared to that in non-RCC cell lines, GET4 had a preferential role as a tumor dependency gene in RCC, and it seemed that microsatellite instability or tumor type did not affect the functions of GET4 across the cell lines. CRB3 appeared to be a potential common tumor suppressor gene according to the RNAi screening results in which most gene effect scores were positive, and RCC lineage, MSI state, and tumor type did not appear to be associated with the effect of CRB3 knockdown. These findings were consistent with our former evaluation of GET4 and CRB3 in the KIRC cohort for survival analysis in the present study. In addition, peptide chain A of the GET4 protein was found to have a ligandable structure (data not shown) in a drug discovery knowledgebase (canSAR), and this structure could be utilized as a therapeutic target in the future.

Furthermore, we validated the expression levels of CRB3 and GET4 in three RCC cell lines (786-O, SW839, A498) and HEK 293T (Supplementary Figure 7). Then data was collected from HPA database (https://www.proteinatlas.org/) showing that GET4 was moderately (7/11) and broadly (7/11, >75% quantity) expressed in RCC tissues while CRB3 was almost negatively (12/12) expressed in RCC tissues but strongly expressed in normal renal tissue. Further validation study of these prognostic genes from patients' samples could be critical and should be considered in the future.

## Discussion

Large-scale investigation with RNAi-based or CRISPR/Cas9-based loss-of-function screening provides an unprecedented landscape of how human cancer cell lines maintain the fundamental features of immortality and whether the proliferation and survival of these cells are dependent on a specific gene's existence or expression. Most human tumors harbor numerous genetic alterations, and some of them are considered drivers or passengers of tumorigenesis 21. Furthermore, these alterations could also cause genotype-specific vulnerabilities across cancer types when compared to normal tissues because multiple changes, including genetic and epigenetic mechanisms, are prerequisites for programming the malignant state 4. Genome-scale screens for potential dependency genes, such as DepMap, provide a wealth of resources for research, and hundreds of candidate genes need further evaluation as available therapeutic targets. However, it is undeniable that translation of evidence from cancer cell lines to clinical application has a long way to go. Moreover, the prognostic value of these potential dependency genes requires further validation in a real-world patient cohort, and differences between the heterogeneous tumors in patients and much more homogenous characteristics of cell lines should be noted. The latest study has revealed that 3D cancer spheroid models could be a better tool for CRISPR screens for potential subtype-specific vulnerabilities since the effect of a gene knockout would vary under different cultivation conditions, and phenotypes observed in 3D models would more accurately simulate and recapitulate the situation that occurred *in vivo* 22. Thus, research focusing on the actual prognostic roles of these genes is needed 23, 24. In the present study, the top preferential dependency genes in ccRCC cell lines from the DepMap database were collected, and their characteristics and correlations with prognoses were further investigated through TCGA datasets and multiple other open platforms.

In total, 16 genes were selected from the greatest preferential gene lists of ccRCC cell lines. Among these genes, PAX8 and HNF1B are the most common dependency genes across ccRCC cell lines and were previously classified as lineage-specific dependency transcription factors, and elevated expression levels predicted their roles as dependency genes 4. Additionally, PAX8 in ovarian cell lines and HNF1B in colorectal cell lines showed similar enrichment tendencies 4. However, the gene effects of PAX8 and HNF1B have a wide range across cell lines, even in the same histological type (Fig. 1), and this diversity may be partially explained by their expression-dependent features; only in a highly expressed context could they show cell line dependency properties. The expression of PAX8 and HNF1B across normal tissues also supported their tissue specificity, and the extremely high expression of PAX8 in the thyroid could indicate its potential dependency role in thyroid cancer. Comparison between unpaired and paired tumor and adjacent normal tissues from the KIRC cohort revealed that the expression of PAX8 and HNF1B was downregulated in tumor tissues, which might be different from some ccRCC cell lines. The genetic alteration rates of PAX8 and HNF1B in TCGA ccRCC dataset were 6% and 7%, respectively (Fig. 4), and these figures might not reach the threshold due to their mutation- or expression-based dependency quality. As expected, these two genes did not show predictive value for OS in ccRCC patients from the KIRC cohort. TNFSF10 and BCL2L1 were other selected genes that were considered expression-driven dependency genes 4, and TNFSF10 belonged to the family of tumor necrosis factor (TNF) ligands that could induce apoptosis in tumor cells but normally did not kill healthy cells 25. TNFSF10 was broadly expressed across human normal tissues (Fig. 2) and was upregulated in tumor tissues according to the comparison results in the present study (Fig. 5). Additionally, high expression of TNFSF10 was a positive predictor for both longer OS and DFS in the KIRC cohort, which might be associated with its original function as a tumor necrosis factor. Furthermore, TNFSF10 was positively associated with several immune cells, including B cells, CD8+ T cells, macrophages, neutrophils, and dendritic cells. BCL2L1 is a protein-coding gene related to apoptosis regulation, and its alternative splicing results in two different isoforms in which the shorter isoform acts as an apoptosis activator and the longer isoform acts as an inhibitor 26. However, the KM curves in our study revealed that a high expression level of BCL2L1 could be a favorable prognostic factor in both the OS and DFS cohorts, and there was no significant difference between the expression of BCL2L1 in tumor and normal tissues, suggesting that not only expression but also alternative splicing could play crucial roles in the influence of a certain gene related to patient survival.

In addition to these expression-driven dependency genes, other genes, such as FERMT2 and COPG1, belonged to a special category called paralog deficiency, in which dependency was triggered by the functional loss of another paralog 4. For example, solid tumor lineages often express FERMT2, and in the cell line subsets without FERMT2 expression, FERMT1 became exquisitely important for cell survival 4. Interestingly, compared to those in other organs, FERMT2 and COPG1 in normal kidney tissue were only moderately expressed, and their gene effect ranges were quite different between the RNAi and CRISPR/Cas9 screening groups in ccRCC cell lines. This might be caused by the intrinsic differences between the two methods, and the degree of loss-of-function achieved by these two methods in a specific gene would obviously vary. These two genes had a low genetic alteration rate of 1.1% for FERMT2 and 0.7% for COPG1 in the KIRC cohort. More importantly, FERMT2 was found to be significantly underexpressed in tumor tissues, and COPG1 was slightly overexpressed in tumor tissues; thus, it was expected that FERMT2 was a positive predictor for overall survival and that COPG1 was a risk factor for disease-free survival. Moreover, we evaluated expression of FERMT2, FERMT1, COPG1, and COPG2 in the KIRC cohort through the GEPIA2 website (data not shown) and found that the median expression values of FERMT2 and COPG1 were higher than those of FERMT1 and COPG2, but the detailed relationships and prognostic values of these paralogs in ccRCC patients need further exploration.

In addition, among these selected genes, MDM2 is a well-known oncogene that can promote tumor formation by targeting tumor suppressor proteins and by inhibiting p53; therefore, blocking this interaction may be a new potential cancer therapy 27. MDM2 had a relatively low but stable expression among multiple organ systems according to the results from the GTEx dataset in our study, and it was significantly highly expressed in the tumor tissues of ccRCC patients (TCGA dataset). Although MDM2 represented a dependency gene for most ccRCC cell lines in both the RNAi and CRISPR/Cas9 groups according to its gene effect scores, it should be mentioned that a former study advocated that its dependency was directly associated with the genetic features of mutated TP53^7^. Since the mutation of TP53 was not common (approximately 2.6%) in ccRCC cases from the TCGA dataset 2 and the mutation rate of MDM2 itself was also low (Fig. 4), it was understandable that it did not show prognostic value for either OS or DFS. CDH2 is another gene significantly overexpressed in tumor tissue, and it is a member of the cadherin superfamily, which has been validated to have a key role in epithelial-to-mesenchymal transition (EMT) and metastasis for nearby and distant breast cancer tissues 28. However, CDH2 was shown to be a favorable prognostic factor in KIRC and a prognostic risk factor in brain lower-grade glioma (LGG) and mesothelioma (MESO). Notably, these 16 genes showed almost completely different prognostic values in LGG compared to those in KIRC.

Based on the multivariable Cox models, GET4 and CRB3 were independent prognostic factors after adjusting for tumor stage and patient age and sex. Previous studies have revealed that CRB3 is a tumor suppressor in mammalian (murine) cells and a favorable prognostic factor in RCC patients from a population cohort of 136 people using tissue microarrays as analysis resources^29^, ^30^. Data from TCGA dataset in our study also confirmed this finding, and evidence from our study showed that CRB3 might be a broad-spectrum tumor suppressor gene according to its positive gene effect scores across different cell lines. It also suggested that maintaining cellular polarity would be critical for maintenance of cell proliferation. Theoretically, the high CRB3 expression in normal tissues (except kidney), such as stomach, lung, and skin, that indicates its potential protective function could also exist in cancer from these tissue sites, but this conclusion needs further assessment and validation in the future. In contrast, GET4 (Golgi to ER traffic protein 4 homolog) showed preferential ccRCC dependency among different lineages of cell lines, and the correlation between GET4 and cancer has been poorly studied. The conserved GET pathway (GET1-5) is considered a transportation mediator of tail-anchored proteins to the endoplasmic reticulum 31. GET4-mediated regulation of ATP hydrolysis has been proven to be an essential step for efficient tail-anchored protein targeting based on biochemical structure analysis 32, 33. In general, a dependency gene validated in a cell line did not directly represent its role in patients. First, different types of dependencies, their corresponding genetic alterations and their expression in normal, adjacent normal, and tumor tissues should be considered. Second, although these effects derived from screening results have been normalized against the distribution of pan-essential and nonessential genes and showed a strong preferential lineage tendency, they can be only ambiguously predicted in real patients with the same histological type. Third, if we start to consider the target tractability as druggable therapeutic resources, it should be more carefully screened for potential candidates. Of note, GET4 showed a ligandable structure in the canSAR database whose links were provided by DepMap on the detailed page of the specific gene.

In summary, 16 genes were selected from the 106 top ccRCC lineage preferential dependency candidates derived from DepMap, an integrated large-scale RNAi and CRISPR/Cas9 screening project of hundreds of human cell lines; these genes included B4GALT4, BCL2L1, CDH2, COPG1, CRB3, FERMT2, GET4, GPX4, HNF1B, ITGAV, MDM2, NFE2L2, PAX8, RUVBL1, TFRC, and TNFSF10. Functional analysis indicated that these genes were enriched in the apoptotic signaling pathway. However, the prognostic value of these signature genes might be dependent on multiple factors, including driving types, genetic alteration rates and expression levels. According to the multivariable Cox regression model, GET4 and CRB3 were independent prognostic factors of OS in the KIRC cohort after adjustment for critical clinical variables. Furthermore, CRB3 seemed to be a potential broad tumor suppressor gene, while GET4 might be a ccRCC preferential dependency gene with a ligandable structure that could facilitate future drug development.

## Supplementary Material

Supplementary figures and tables.

## Figures and Tables

**Table 1:** Univariable and multivariable COX proportional hazard model

Variable	P-value	HR	Lower 95%CI	Upper 95%CI	P-value	HR	Lower 95%CI	Upper 95%CI
Gender	0.759	0.952	0.697	1.301				
Stage II vs Stage I	0.473	1.255	0.675	2.335				
Stage III vs Stage I	<0.001*	2.648	1.754	3.997	0.001*	2.057	1.355	3.124
Stage IV vs Stage I	<0.001*	6.730	4.585	9.879	<0.001*	5.518	3.707	8.214
Age	<0.001*	1.029	1.016	1.043	<0.001*	1.032	1.017	1.047
TNFSF10	0.307	1.000	0.999	1.000				
TFRC	0.607	1.002	0.995	1.009				
RUVBL1	0.991	1.000	0.985	1.016				
PAX8	0.339	1.003	0.997	1.008				
NFE2L2	<0.001*	0.991	0.986	0.995				
MDM2	0.017	0.988	0.979	0.998				
ITGAV	<0.001*	0.992	0.988	0.996				
HNF1B	0.105	0.997	0.994	1.001				
GPX4	0.599	1.000	0.999	1.001				
GET4	0.012*	1.021	1.004	1.037	0.002*	1.023	1.009	1.038
FERMT2	0.001*	0.986	0.977	0.994				
CRB3	<0.001*	0.960	0.950	0.970	<0.001*	0.969	0.960	0.980
COPG1	0.923	1.000	0.995	1.005				
CDH2	0.015*	0.992	0.985	0.998				
BCL2L1	0.012*	0.995	0.991	0.999				
B4GALT4	0.851	0.999	0.984	1.014				

* represent p-value<0.05, HR, hazard ratio, CI, confidence interval
