# Supplementary material for: Characteristics and prognostic value of potential dependency genes in clear cell renal cell carcinoma based on a large-scale CRISPR-Cas9 and RNAi screening database DepMap
Source: Int J Med Sci. 2021 Mar 11;18(9):2063–75. doi: 10.7150/ijms.51703 (PMC8040392; doi:10.7150/ijms.51703)

Supplementary Table 1. Correlation between expression, relative copy number and methylation of selected genes

Gene	Correlation between expression and relative copy number (Spearman coefficient)	Correlation between expression and methylation (Spearman coefficient)
B4GALT4	0.42	-0.28
BCL2L1	0.17	-0.18
CDH2	0.25	-0.37
COPG1	0.51	NA
CRB3	0.13	-0.47
FERMT2	0.42	-0.38
GET4	0.34	NA
GPX4	0.24	-0.28
HNF1B	0.15	-0.16
ITGAV	0.14	-0.27
MDM2	0.14	-0.13
NFE2L2	0.19	-0.09
PAX8	0.14	-0.27
RUVBL1	0.39	-0.34
TFRC	0.21	-0.01
TNFSF10	0.10	-0.43

NA, not available

Supplementary Figure 1 Flow diagram of data acquisition and analysis

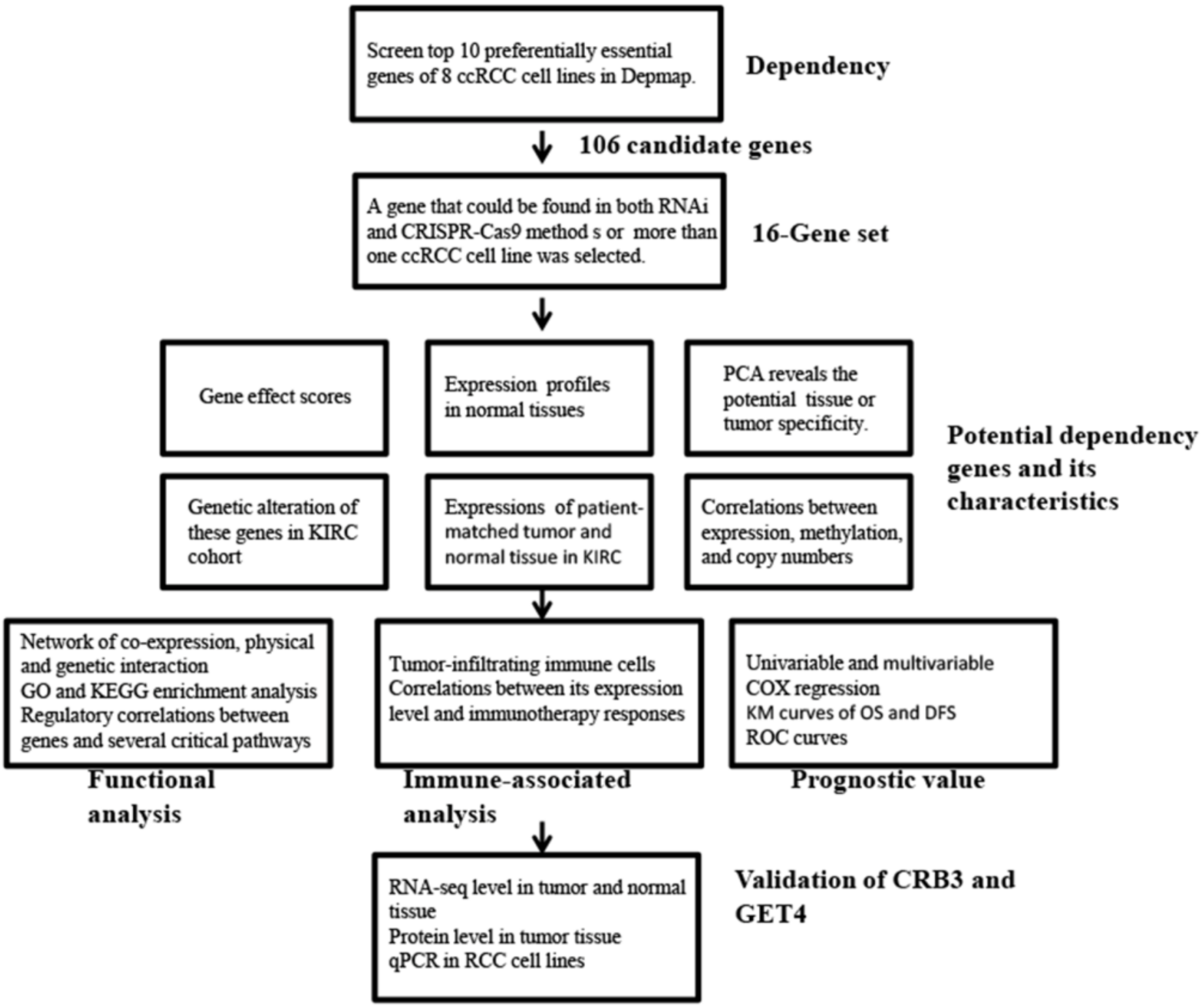

Supplementary Figure 2. Expression of genes in tumor and unpaired adjacent normal tissue in the KIRC cohort

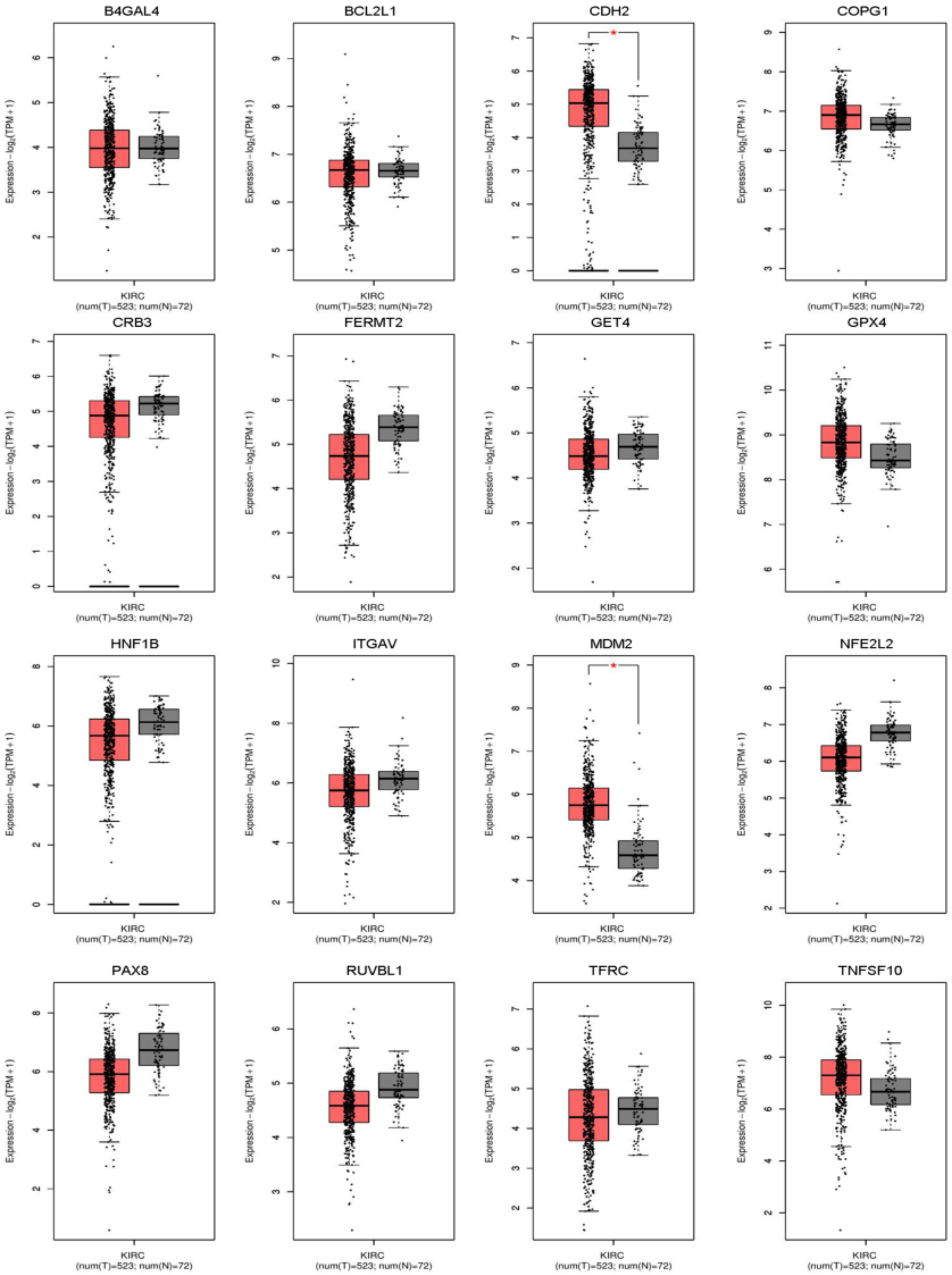

Supplementary Figure 3. Functional analysis

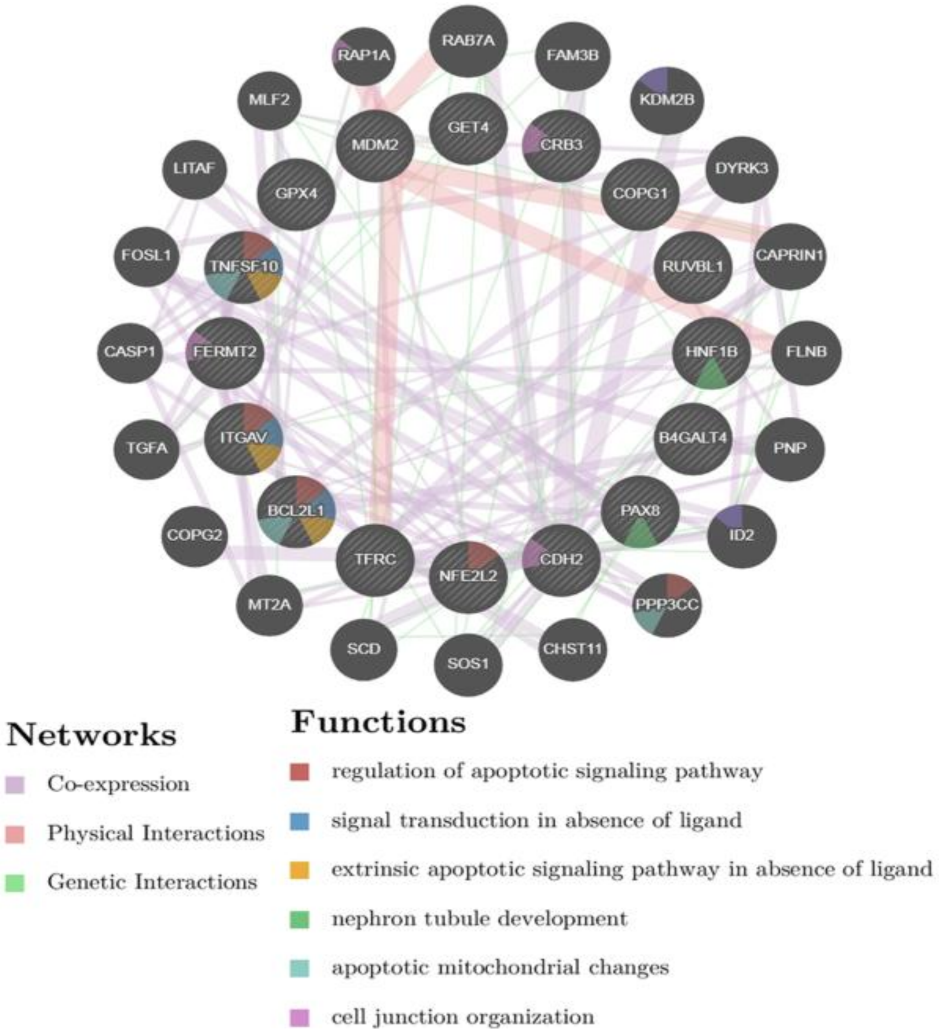

Supplementary Figure 4. Differential expression between responders and non-responders from two datasets

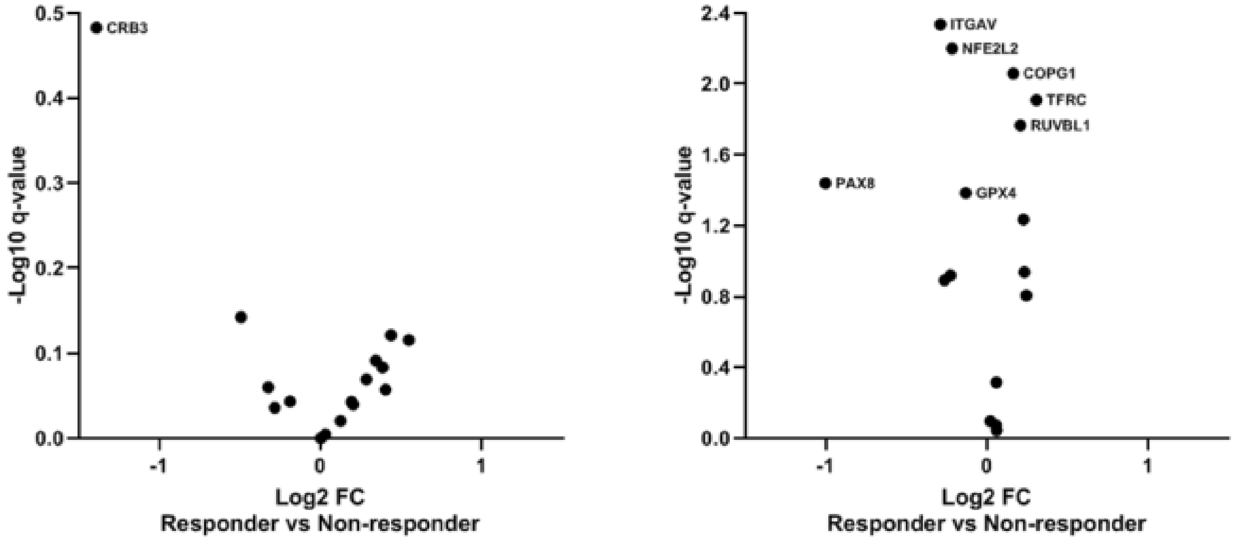

Supplementary Figure 5. ROC curves

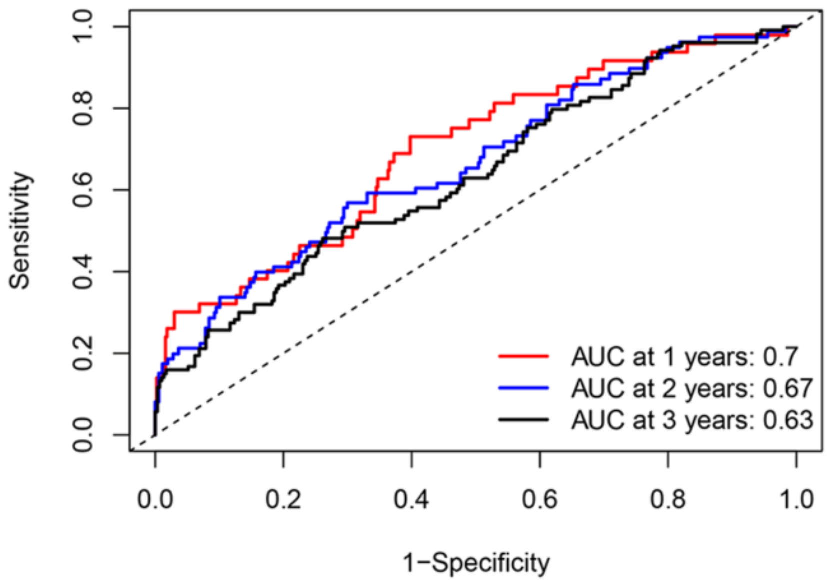

Supplementary Figure 6. Gene effect scores of GET4 and CRB3 between different lineages, microsatellite status, and tumor types of cell lines

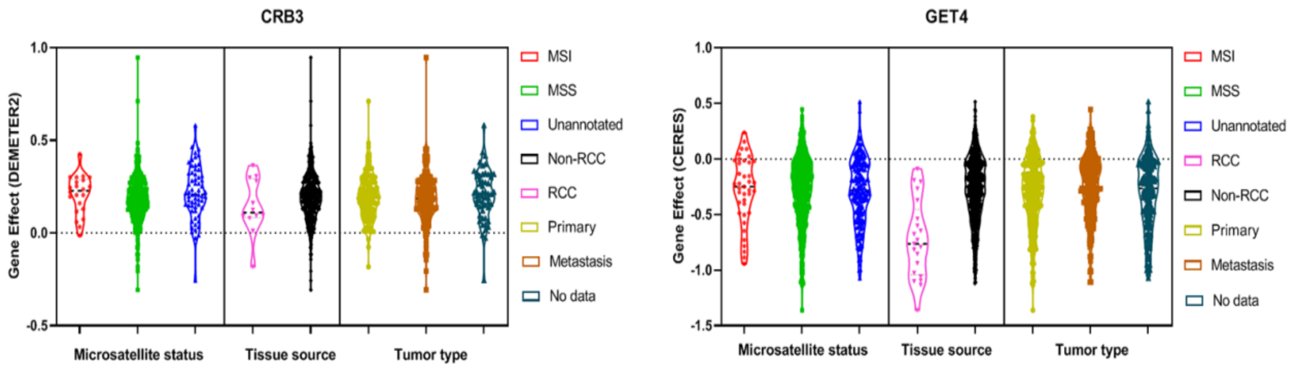

Supplementary Figure 7. Expression levels of CRB3 and GET4 in cell lines (qPCR)

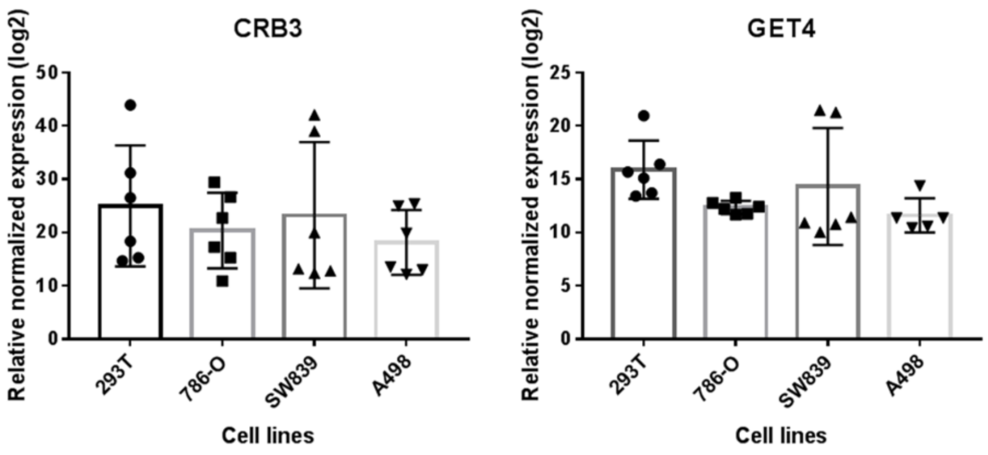

Supplement: Supplementary file 1 — Supplementary figures and tables. [file ijmsv18p2063s1.pdf]
